# Aseptic meningitis in multisystem inflammatory syndrome in children associated with coronavirus disease 2019: a case report

**DOI:** 10.1186/s13256-022-03617-y

**Published:** 2022-10-04

**Authors:** Alije Keka-Sylaj, Atifete Ramosaj, Arbana Baloku, Qëndresa Beqiraj, Petrit Gjaka

**Affiliations:** 1 Institute of Anatomy, Pediatric Clinic, Faculty of Medicine, University of Prishtina, Pristina, 10000 Kosovo; 2 Pediatric Clinic, University Clinical Center of Kosovo, Pristina, 10000 Kosovo

**Keywords:** MIS-C, COVID 19, Children, Aseptic meningitis

## Abstract

**Background:**

As the coronavirus disease 2019 infections are still ongoing, there is an increasing number of case reports and case series with various manifestations of life-threatening multisystem inflammatory syndrome in children . Our case aims to remind all providers to scrutinize for clinical manifestations, including neurological symptoms, which may mimic aseptic meningitis.

**Case presentation:**

A 5-year-old Albanian male child with obesity was admitted to the pediatric intensive care unit due to persistent fever, headache, vomiting, abdominal pain, mucocutaneous manifestations, and fatigue. Initial laboratory results revealed high level of inflammatory markers, including C-reactive protein of 156.8 mg/l, erythrocyte sedimentation rate of 100 mm/hour, procalcitonin of 13.84, leukocytosis with neutrophilia, and lymphopenia. Liver and renal functions, and capillary blood electrolytes (Na, K, Ca), were also altered. Cerebrospinal fluid was slightly turbid, with a white blood cell count of 128/mm^3^ (80% mononuclear cells and 20% polymorphonuclear), consistent with aseptic meningitis. The clinical presentation with prolonged fever, multiorgan dysfunction, and elevated inflammatory markers, with no plausible alternative diagnosis, matches the case definition of multisystem inflammatory syndrome in children. Combining corticosteroid methylprednisolone with intravenous immunoglobulin was effective.

**Conclusions:**

Apart from the most common presentation of multisystem organ dysfunction, neurological manifestations of multisystem inflammatory syndrome in children such as aseptic meningitis, may be present as an immune response post-viral to coronavirus disease 2019. Given the rapid deterioration of children with multisystem inflammatory syndrome, early treatment with immunoglobulins and corticosteroids should be considered.

## Introduction

As in many places around the world, the ongoing coronavirus disease 2019 (COVID-19) pandemic has quickly swept Kosovo since it started in March 2020. Compared with adults, children and adolescents are generally at a lower risk of infection, and most of them manifest asymptomatic or mild forms of the disease [1]. However, some children and young people have experienced severe symptoms of the disease, with some requiring hospitalization, and a few have died [1]. As of late April 2020, there have been increasing reports of a new and potentially life-threatening childhood disease that has been temporally associated with the severe acute respiratory syndrome coronavirus 2 (SARS-CoV-2) infection, manifesting with fever and multisystem organ involvement due to hyperinflammation [2].

This disease, with features commonly seen in both Kawasaki disease (KD) and toxic shock syndrome, was named multisystem inflammatory syndrome in children (MIS-C) by the Centers for Disease Control and Prevention (CDC) and the World Health Organization (WHO) [3, 4].

The pathophysiology and clinical course of this new SARS-CoV-2 strain in children are still unknown [5]. However, the definition of the largest number of cases is based on evidence of the SARS-CoV-2 infection or exposure through close contact with an individual with confirmed COVID-19, pediatric age, persistence of fever, elevated inflammatory markers, and the manifestation of multiorgan dysfunction, as well as the lack of an alternative diagnosis [6].

We present the disease course of a 5-year-old male child with obesity, who was admitted to the intensive care unit of the pediatric clinic in a critical condition.

## Case presentation

**Fig. 1:**
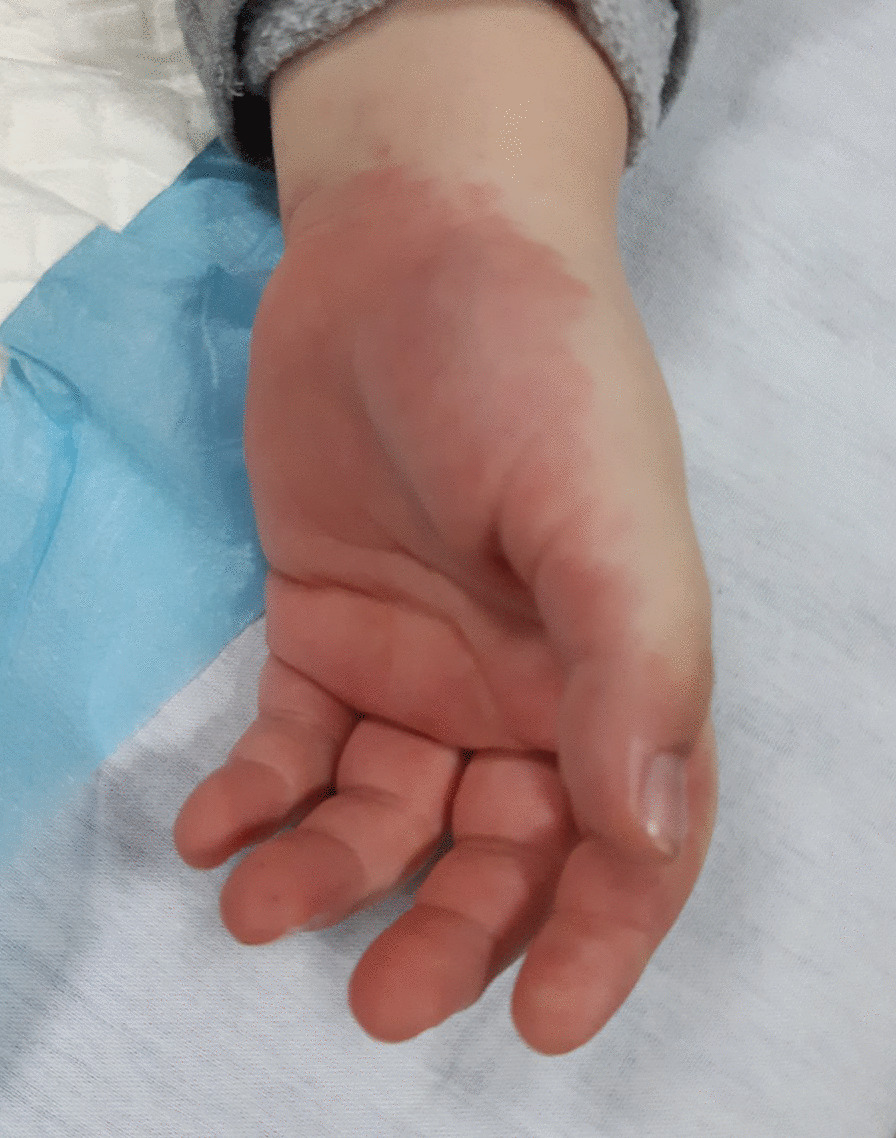
Palmar erythema on the first day of hospitalization

**Fig. 2:**
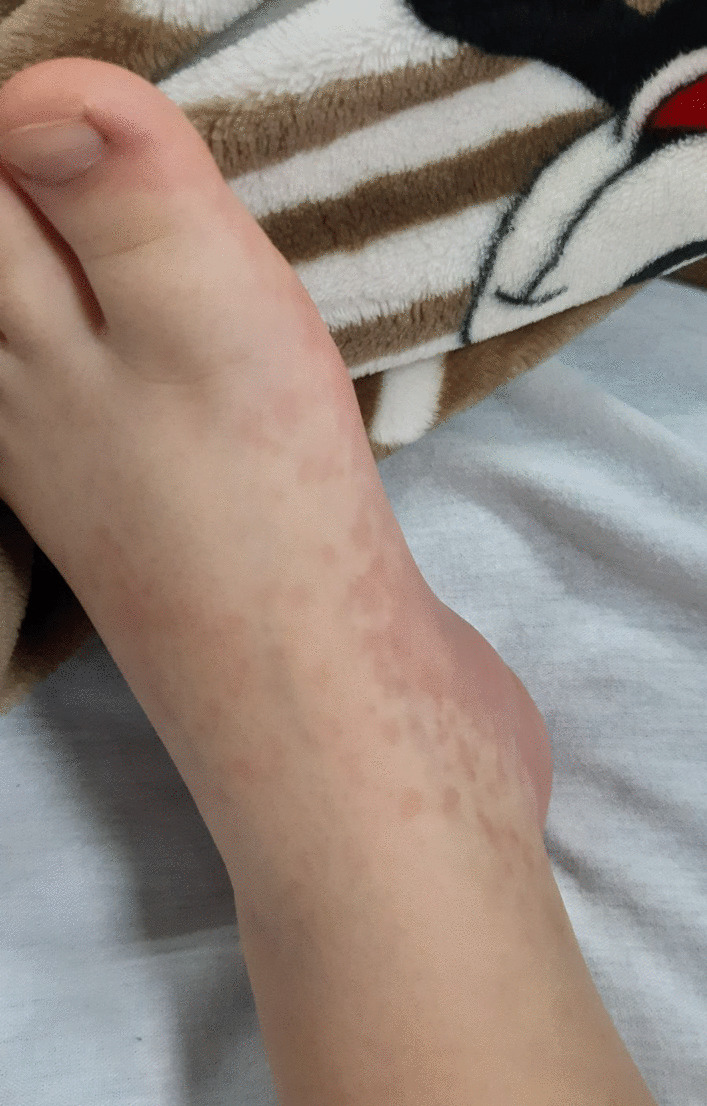
Redness and swelling from the upper surface to the sole of the foot

We report a critically ill patient, a 5-year-old Albanian male, presenting with features of MIS-C in January 2021, in the pediatric intensive care unit of the University Clinical Center of Kosovo. The initial symptoms appeared 3 weeks prior to admission, consisting of fatigue, occasional headache, and loss of appetite. Meanwhile, 1 week before hospitalization, fever appeared up to 40 °C, recurring every 4–6 hours. He had previously been treated for a nasopharyngeal inflammation with antipyretics and antibiotics. However, the fever continued, and the illness progressed with vomiting and abdominal pain, fatigue, and refusal to eat. The day before admission at the intensive care unit, his family became concerned by a new onset of bilateral eye redness, swelling, redness of hands and feet, rash, irritability, and photophobia. At admission, he had fever of up to 40 °C, arterial hypotension with blood pressure (BP) of 70/40 mmHg, tachycardia with a heart rate (HR) of 130/min, tachypnea with a respiratory rate (RR) of 45/minute, oxygen saturation (SpO_2_) of 93%, paleness, and dehydration, with a chest capillary refill time (CRT) of 5 seconds. Signs of hyperinflammatory syndrome were also present, with mucocutaneous inflammation signs such as swollen and red eyelids, bilateral nonpurulent conjunctivitis, oral mucosal lesions, dry lips, strawberry-red tongue, and oropharyngeal hyperemia. There was no cervical lymphadenopathy. Redness and swelling were also present on the palms of the hands and the soles of the feet (Figs. 1 and 2).

The child’s mental status and level of consciousness were also altered. He was lethargic and irritable, with photophobia during the examination. His lungs were clear and had no murmurs or cardiac friction rub. The abdomen was soft without hepatosplenomegaly, but was painful during palpation. Based on a height of 120 cm and a weight of 34 kg, he had obesity with a body mass index (BMI) of 23.6, placing his BMI for age at greater than the 99th percentile.

There was a history of positive COVID-19 contact 3 weeks ago when his uncle had a brief febrile upper respiratory infection and was tested and confirmed positive for SARS-CoV-2 with a polymerase chain reaction (PCR) test. Meanwhile, his asymptomatic father had a negative PCR test for SARS COV-2 but positive serology with a high immunoglobulin G (IgG) antibody to SARS COV-2 on the day of the child’s admission. The child remained at home with his immediate family during this period but was not tested for SARS COV-2 infection. There was no personal or family history of allergic reactions, vasculitis, autoimmune disorders, cardiac disease, diabetes, or hereditary disease. This was the third hospitalization of the child; the first one at the age of 2 due to gastroenteritis; and the second one, at the age of 4, due to a urinary tract infection.

Initial laboratory results revealed a high level of inflammatory markers, including a C-reactive protein (CRP) of 156.8 mg/l, an erythrocyte sedimentation rate (ESR) of 100 mm/hour, procalcitonin (PCT) of 13.84 (Table 1), leukocytosis with neutrophilia, and lymphopenia (Table 2). Liver and renal functions were also altered, manifested by hypoalbuminemia (30.6 g/L), hypoproteinemia (60.3 g/L), hyperuricemia (602.77 umol/L), uremia (15.81 mmol/L), and an increased level of serum creatinine (114 umol/L) as a consequence of renal dysfunction (acute kidney injury), which resolved with hydration (Table 1). There was a mild elevation of cholesterol and significant elevation of triglycerides (5.29 mmol/L) and gamma-glutamyltranspeptidase (GGT, 79 U/L) with normal alanine aminotransferase (ALT), aspartate aminotransferase (AST), alkaline phosphatase (ALP), lactic dehydrogenase (LDH), and creatine kinase (CK) levels (Table 1). The capillary blood electrolytes (Na, K, Ca) were also altered, with hyponatremia (129 mmol/L), a low level of bicarbonates (19.7 mmol/L), a base excess (BE) of 4 mmol/L, and hypoxemia with capillary blood gas of pO_2_ of 63 mmHg and pCO_2_ of 29 mmHg (Table 3). A lumbar puncture was performed owing to fever, irritability, altered level of consciousness, and photophobia.The cerebrospinal fluid (CSF) was slightly turbid, with a white blood cell (WBC) count of 128/mm^3^ (80% mononuclear cells and 20% polymorphonuclear), CSF protein of 1.14 g/L (normal range 0.10–0.45 g/L) and CSF glucose of 3.84 mmol/L (simultaneous blood glucose level of 5.45 mmol/L). The bacterial culture of CSF was negative. The findings from the CSF examination were consistent with aseptic meningitis. Normal fundus findings were found on ophthalmoscopy examination . Nasopharyngeal SARS-CoV-2 PCR testing was negative, while SARS-CoV-2 IgG testing was positive. D-dimer was high, at 464 ng/mL and 833 ng/ml by two measurements (reference range ˂ 200), while the coagulation tests were within the normal range (Table 1). The urinalysis revealed proteinuria (30–100 mg/dl), which was confirmed by 24-hour urine collection. Leukocyturia and occasionally bacteriuria were also present, but the urine culture was negative. Moreover, no other obvious microbial cause of inflammation was found, including two blood cultures for bacterial sepsis, three urine cultures, and nasopharyngeal and CSF cultures.

**Table 1:** Laboratory values and trends during hospital stay

Parameters	Day 1	Day 2	Day 4	Day 7	Day 11	Day 14
SE (5–10)	100			75		40
CRP (0.0–6.0 mg/L)	156.8	116.8	30.1	19.8	32.7	16.7
PCT (0.0–0.5 ng/ml)	13.84	6.73		0.578		0.2
Chol (3.60–5.70 mmol/L)	6.0	4.62		4.27	5.11	5.22
Trig (0.45–1.81 mmol/L)	5.29			3.05	2.89	2.04
GGT (3–55 U/L)	74		34			30
Urea (1.70–8.30 mmol/L)	15.81	10.37	7.74	11.5	6.48	6.84
Uric Ac (155–430 umol/L)	602.7					310
Creat (53–115 umol/L)	114	60.0	71.4	92.5	71	61.2
Albumin (35.0–52.0 g/L)	30.6	33.6	32.8	35.1	38.1	36
TP (64.0–83.0 g/L)	60.3	60.5		67.4	73.5	68.7
D-dimer (˂ 200)		464	833	653	450	311
ALT (3–41 U/L)	36	29	13	11	13	8
AST (2–37 U/L)	39	31	19	22	19	16
Amylase (27–102 U/L)	37					
ALP (43–115 U/L)	179					85
GGT (3–55 U/L)	74		34			
CK (38–171 U/L)	20	46	21			10
LDH (230–460 U/L)	460	465	439	482		342
Ca (2.10–2.55 mmol/L)	2.14		2.13			
P (0.90–1.50 mmol/L)	1.82					1.1
Fe (11.6–31.3 umol/L)	2.6		5.3	5.5	13.2	

CRP: C-reactive protein; ESR: erythrocyte sedimentation rate; PCT: procalcitonin; Chol: cholesterol; Trig: triglycerides; Creat: creatinine; TP: total protein; ALT: alanine aminotransferase; AST: aspartate aminotransferase; ALP: alkaline phosphatase; GGT: gamma-glutamyltranspeptidase; CK: creatine kinase; LDH: lactic dehydrogenase; Ca: calcium; P: phosphorous; Fe: iron

**Table 2:** Complete blood cells count (CBC) and trends during hospital stay

Parameters	Day 1		Day 2	Day 3	Day 5	Day 7	Day 14
WBC (3.5–10)	18.6	15	11.4	16.6	15.3	14.2	13
RBC (3.8–5.8)	4.32	3.86	2.19	4.29	2.77	4.03	3.41
HGB (11.0–16.5)	10.9	10.4	84	10.9	10.6	10.7	10.6
HCT (35.0–50.0)	30.9	22.7	17.2	34.8	22.6	32.7	27.5
PLT (150–390)	183	106	116	202	174	445	370
LYM (17.0–48.0%)	9.10	10.5	10.3	13.4	11.5	25.2	48.7
GRA (43.0–76.0%)	88.5	86.2	86.5	83.2	85.3	70.2	45.5

WBC: white blood cells; RBC: red blood cells; HGB: hemoglobin; HCT: hematocrit; PLT: platelets; LYM: lymphocytes; GRA: granulocytes

**Table 3:** Capillary blood gases and electrolytes

Parameters	Day 1		Day 2	Day 3	Day 6	Day 8	Day 11
Ph (7.35–7.45)	7.47	7.46	7.56	7.49	7.53	7.45	7.4
pCO_2_ (34–46 mmHg)	27	33	24	35	34	39	32
pO_2_ (80–105 mmHg)	51	51	98	57	68	54	74
Na^+^ (130–145 mmol/L)	129	130	131	134	139	137	135
K^+^(3.4–5.1 mmol/L)	4.5	3.8	3.5	3.5	3.5	2.6	3.5
Ca^++^(1.17–1.24 mmol/L)	1.15	1.21	0.95	1.17	1.15	1.19	1.19
HCO_3_ (22–26 mmol/L)	19.7	23.5	21.5	26.7	28.4	27.1	25.3
BE (−4 to +2 mmol/L)	−4	−0.3	−0.7	3.4	5.7	3.1	2.2

Ph: acid-base balance of the blood; pCO_2_: partial pressure of carbon dioxide; pO_2_: partial pressure of oxygen; Na: sodium; K: potassium; Ca: calcium; HCO_3_: bicarbonate; BE: base excess.

**Fig. 3:**
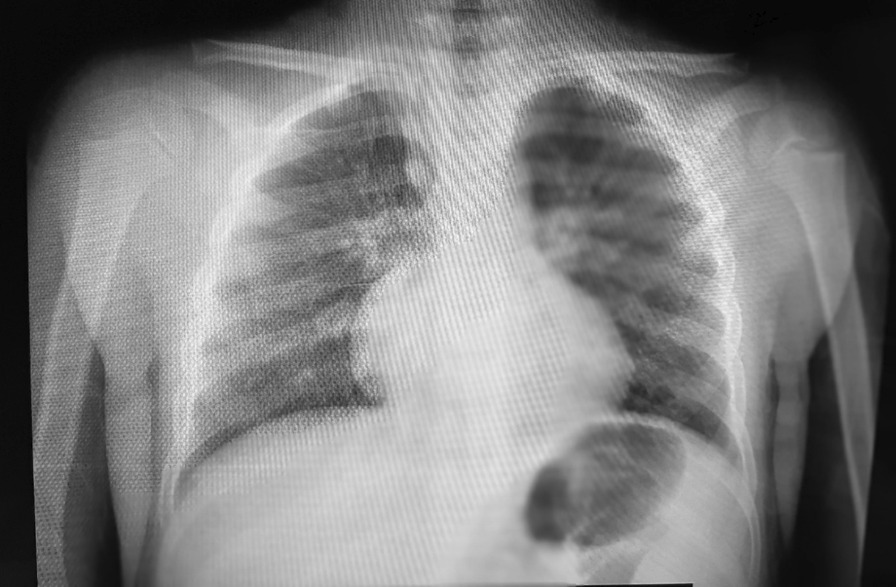
Frontal chest radiography shows bilateral minimal peripheral patchy opacities. The lung ultrasonography showed basal B lines, minimal bilateral basal pleural thickening, and minimal pleural effusion in both phrenicocostal sinuses

The chest radiography showed bilateral minimal peripheral patchy opacities but no focal consolidation, effusion, or pneumothorax (Fig. 3).

The electrocardiogram demonstrated sinus tachycardia with no other pathologic findings. Furthermore, echocardiography and abdominal ultrasonography examinations were normal at admission as well as during the follow-up examination. At the moment of admission, the child was put on oxygen therapy through a nasal cannula with an O_2_ flow of 2–3 L/min. Owing to hypotension and dehydration, rehydration with intravenous fluids, fresh plasma, and packed red blood cells was administered. Treatment also involved antibiotics, including ceftriaxone (80 mg/kg/day) and vancomycin (40 mg/kg/day), methylprednisolone (1 mg/kg/day), and intravenous immunoglobulin (IVIG)(1 g/kg), as well as anticoagulation therapy with enoxaparin.

The fever resolved on the third day of hospitalization, 24 hours after immunoglobulin administration. On the second day of hospitalization, CSF analysis revealed a decreased WBC count of 21/mm^3^ (90% mononuclear cells and 10% polymorphonuclear), 1.05 g/L CSF protein (normal range: 0.10–0.45 g/L) and 4.76 mmol/L CSF glucose (simultaneous blood glucose level: 4.87 mmol/L). In the meantime, the second bacterial culture of CSF was also negative. On the seventh day of hospitalization, the level of albuminemia was 35.1 g/L, CRP 19.8 mg/L, ESR 75 mm/hour, PCT 0.578 ng/ml, D-dimer 653 (Table 1), and white blood cell count of 16.1 (26.7% lymphocytes and 65% granulocytes) (Table 2). The mucocutaneous inflammation signs such as redness of eyelids and conjunctival hyperemia, and the palmar and plantar erythema, significantly faded on the fifth day of hospitalization, but were easily visible until the end of hospitalization. However, the general condition of the child continued to be stable and follow-up examinations such as the electrocardiogram (ECG), echocardiography, lung, and abdominal ultrasound were normal. At the same time, almost all the performed laboratory analyzes were within the normal range (Tables 1, 2, 3), and the child was discharged 2 weeks after admission. During a follow-up outpatient visit, blood tests had normalized, while the nCOV-2 IgG was elevated to 100 (reference range: 1.0). Abdominal, pulmonary, and cardiac ultrasound were normal. Follow-up outpatient visits were carried out every 3 months for 1 year, including echocardiography, urine analysis, and evaluation of his general condition.

Apart from proteinuria which declined week by week but persisted for 2 months, other findings were normal.

## Discussion

Our case is of particular interest because it shows that there is a clinical variety of symptoms, including neurological symptoms, as an immune response post-viral to COVID-19 infection, even if the RT-PCR test for SARS-CoV-2 from the patient’s nasopharyngeal specimen was negative.

The COVID-19 pandemic is still ongoing, with unprecedented consequences for the health of millions of people and causing thousands of deaths worldwide [7]. Even though the risk of severe disease and death is highest in older people and persons with underlying noncommunicable diseases (NCDs), recent reports are presenting that some children do require hospitalization and intensive care due to a new and potentially life-threatening childhood disease, referred to as MIS-C [8]. Unfortunately, child fatalities have been reported due to COVID-19, from MIS-C [6, 9].

MIS-C typically manifests 3–4 weeks after SARS-CoV-2 infection; therefore, many children had positive antibodies to SARS- CoV-2 but negative nasopharyngeal SARS-CoV-2 PCR tests at the time of MIS-C evaluation [10, 11]. This suggests that this inflammatory syndrome is not mediated by a direct viral invasion, but coincides with the development of immune responses acquired as a result of SARS-CoV-2 [12]. Commonly, children with MIS-C have severe forms of the disease, with a history of high fever and a variable number of symptoms due to multiorgan dysfunction of at least four organ systems, most commonly the gastrointestinal, cardiovascular, hematologic, mucocutaneous, and respiratory systems [10]. Recent reports describe emerging cases of MIS-C related to COVID-19 infection, which share common characteristics with Kawasaki disease, Kawasaki disease shock syndrome, toxic shock syndrome, and macrophage activation syndromes [13, 14]. This can be due to a hyperinflammatory response either by antibody-mediated enhancement or by other mechanisms [15, 16].

Our case report focused on a description of the presentation and clinical course of MIS-C in a 5-year-old male child with obesity, who presented with a history of 5 days of fever, elevated inflammatory markers, and multiorgan dysfunction, with no plausible alternative diagnosis, which matches the case definition of MIS-C [17]. The clinical manifestations were similar to toxic shock syndrome, with altered mental status and level of consciousness, moreover, with CSF findings consistent with aseptic meningitis, which is a rare condition, but has also been observed in a few published reports [18–20].

Compared with adults, the incidence of encephalitis or meningitis associated with SARS-CoV-2 is relatively low in children and adolescents (31.25%), which may be related to the relatively mild illness of COVID-19 in this age group [21]. The pathophysiological characteristics of SARS-CoV-2-associated meningoencephalitis are still unclear, but may be related to neuronal cell edema secondary to neuroinflammatory injury, due to cytokine storm syndrome induced by the overreaction of monocytes, macrophages, and T cells after SARS-CoV-2 infection or release of interleukins-6 (IL-6) [18]. Although only a few case reports have presented neurological involvement, they highlight the neurotropism of the SARS-CoV-2 virus [19, 22]. Viral meningitis with positive PCR for COVID-19 in the cerebrospinal fluid without pulmonary involvement has also been described in children and adults [23, 24]. In our case, although the results of the CSF were consistent with aseptic meningitis, we suggest that this was a systemic inflammatory response effect in the central nervous system (CNS). We support this by negative findings in CSF 2 days after the administration of antiinflammatory therapy. Based on clinical presentation, laboratory findings, and additional examinations with no plausible alternative diagnosis, the case was treated successfully with therapy according to clinical guidance for MIS-C associated with SARS–CoV‐2 [25].

## Conclusion

This is a rare case report of MIS-C associated with SARS-CoV-2 infection in a previously healthy male child, who became critically ill with multisystem involvement. Apart from the most common MIS-C presentation of multisystem organ dysfunction, neurological manifestations, such as aseptic meningitis, may be present as an immune response post-viral to COVID-19. Therefore, it should be considered that meningoencephalitis may be a presentation of SARS-CoV-2, even without respiratory symptoms. Given the rapid deterioration of children with MIS-C, early treatment with immunoglobulins and corticosteroids should be considered.

## Data Availability

All of the data and materials will be available from the corresponding author upon request.

## Abbreviations

BP: Blood pressure
CDC: Centers for Disease Control and Prevention
CRT: Chest capillary refill time
CSF: Cerebrospinal fluid
ECG: Electrocardiogram
HR: Heart rate
KD: Kawasaki disease
MIS-C: Multisystem inflammatory syndrome in children
NCDs: Noncommunicable diseases
PCR: Polymerase chain reaction
RR: Respiratory rate
WHO: World Health Organization

## References

[1] She J, Liu L, Liu W (2020). COVID-19 epidemic: disease characteristics in children. J Med Virol.

[2] Multisystem inflammatory syndrome in children and adolescents with COVID-19. World Health Organization. https://www.who.int/publications/i/item/multisystem-inflammatory-syndrome-in-children-and-adolescents-with-covid-19.

[3] Riphagen S, Gomez X, Gonzalez-Martinez C, Wilkinson N, Theocharis P (2020). Hyperinflammatory shock in children during COVID-19 pandemic. Lancet.

[4] Centers for Disease Control and Prevention Health Alert Network (HAN). 2021. Multisystem Inflammatory Syndrome in Children (MIS-C) Associated with Coronavirus Disease 2019 (COVID-19). https://www.cdc.gov/coronavirus/2019-ncov/hcp/pediatric-hcp.html

[5] Cirks B, Geracht J, Jones O, May J, Mikita C, Rajnik M (2021). Multisystem inflammatory syndrome in children during the COVID-19 pandemic: a case report on managing the hyperinflammation. Mil Med.

[6] Ahmed M, Advani S, Moreira A, Zoretic S, Martinez J, Chorath K (2020). Multisystem inflammatory syndrome in children: a systematic review. EClinicalMedicine..

[7] Euro.who.int. 2021. *COVID-19 and Children*. https://www.euro.who.int/en/health-topics/Life-stages/child-and-adolescent-health/covid-19-and-children.

[8] Shekerdemian L, Mahmood N, Wolfe K, Riggs B, Ross C, McKiernan C (2020). Characteristics and outcomes of children with coronavirus disease 2019 (COVID-19) infection admitted to US and Canadian pediatric intensive care units. JAMA Pediatr.

[9] Hoang A, Chorath K, Moreira A, Evans M, Burmeister-Morton F, Burmeister F (2020). COVID-19 in 7780 pediatric patients: a systematic review. EClinicalMedicine..

[10] Feldstein L, Rose E, Horwitz S, Collins J, Newhams M, Son M (2020). Multisystem inflammatory syndrome in U.S. children and adolescents. N Engl J Med.

[11] Godfred-Cato S, Bryant B, Leung J, Oster M, Conklin L, Abrams J (2020). COVID-19–associated multisystem inflammatory syndrome in children—United States, March–July 2020. MMWR Morb Mortal Wkly Rep.

[12] Jiang L, Tang K, Levin M, Irfan O, Morris S, Wilson K (2020). COVID-19 and multisystem inflammatory syndrome in children and adolescents. Lancet Infect Dis.

[13] Consiglio C, Cotugno N, Sardh F, Pou C, Amodio D, Rodriguez L (2020). The immunology of multisystem inflammatory syndrome in children with COVID-19. Cell.

[14] Kabeerdoss J, Pilania R, Karkhele R, Kumar T, Danda D, Singh S (2020). Severe COVID-19, multisystem inflammatory syndrome in children, and Kawasaki disease: immunological mechanisms, clinical manifestations and management. Rheumatol Int.

[15] Nakra N, Blumberg D, Herrera-Guerra A, Lakshminrusimha S (2020). Multi-system inflammatory syndrome in children (MIS-C) following SARS-CoV-2 infection: review of clinical presentation, hypothetical pathogenesis, and proposed management. Children.

[16] McCrindle B, Manlhiot C (2020). SARS-CoV-2–related inflammatory multisystem syndrome in children. JAMA.

[17] Health Department-reported cases of multisystem inflammatory syndrome in children (MIS-C) in the United States. Centers for Disease Control and Prevention. https://www.cdc.gov/mis-c/cases/index.html

[18] Chen T (2020). Neurological involvement associated with COVID-19 infection in children. J Neurol Sci.

[19] Kest H, Kaushik A, DeBruin W, Colletti M, Goldberg D (2020). Multisystem inflammatory syndrome in children (MIS-C) associated with 2019 novel Coronavirus (SARS-CoV-2) infection. Case Rep Pediatrics..

[20] Chiotos K, Bassiri H, Behrens E, Blatz A, Chang J, Diorio C (2020). Multisystem inflammatory syndrome in children during the Coronavirus 2019 pandemic: a case series. J Pediatric Infect Dis Soc.

[21] Huo L, Xu K, Wang H (2021). Clinical features of SARS-CoV-2-associated encephalitis and meningitis amid COVID-19 pandemic. World J Clin Cases..

[22] Pandey M (2021). Acute meningoencephalitis in a child secondary to SARS-CoV-2 virus. Indian Pediatr.

[23] Yousefi K, Poorbarat S, Abasi Z, Rahimi S, Khakshour A (2021). Viral meningitis associated with COVID-19 in a 9-year-old child: a case report. Pediatr Infect Dis J.

[24] Khodamoradi Z, Hosseini SA, GholampoorSaadi MH, Mehrabi Z, Sasani MR, Yaghoubi S (2020). COVID-19 meningitis without pulmonary involvement with positive cerebrospinal fluid PCR. Eur J Neurol.

[25] Henderson L, Canna S, Friedman K, Gorelik M, Lapidus S, Bassiri H (2020). American College of rheumatology clinical guidance for multisystem inflammatory syndrome in children associated with SARS–CoV-2 and hyperinflammation in pediatric COVID-19: version 1. Arthr Rheumatol..

